# Human Papillomavirus Awareness, Vaccine Status, and Risk Factors in Female Emergency Patients

**DOI:** 10.5811/westjem.2019.12.44422

**Published:** 2020-02-24

**Authors:** Lauren A. Walter, Elizabeth Leader, James W. Galbraith

**Affiliations:** * University of Alabama at Birmingham, Department of Emergency Medicine, Birmingham, Alabama; † University of Mississippi Medical Center, Department of Emergency Medicine, Jackson, Mississippi

## Abstract

**Introduction:**

A vaccine targeting high-risk human papillomavirus (HPV) strains can effectively prevent HPV-associated cervical cancer risk. However, many girls and women do not receive the vaccine, more often those impacted by health disparities associated with race and/or socioeconomic status. This same disparate population has also been shown to be at higher risk for cervical cancer. Many of these women also rely on the emergency department (ED) as a safety net for their healthcare. This study sought to gather information pertaining to HPV and cervical cancer risk factors, awareness of HPV and the vaccine, as well as HPV vaccine uptake in female patients presenting to an ED.

**Methods:**

We obtained 81 surveys completed by female ED patients. Demographics included age, race, income, insurance status, primary care provider status, and known cervical-cancer risk factors. Subsequent survey questions explored respondents’ knowledge, familiarity, and attitudes regarding HPV, cervical cancer, and the HPV vaccine, including vaccination uptake rates. We analyzed data using descriptive statistics and Fisher’s exact test.

**Results:**

Approximately one in seven respondents (14.8%) had never previously heard of HPV and 32.1% were unaware of the existence of a HPV vaccine. Minority patients, including those who were Black and Hispanic patients, low income patients, and uninsured and publicly insured patients were less likely to be aware of HPV and the vaccine and likewise were less likely to be offered and receive the vaccine. More than 60% of all respondents (61.3%) had never previously been offered the vaccine, and only 24.7% of all respondents had completed the vaccine series.

**Conclusion:**

Female ED patients may represent an at-risk cohort with relatively low HPV awareness and low HPV vaccine uptake. The ED could represent a novel opportunity to access and engage high-risk HPV populations.

## INTRODUCTION

Human papillomavirus (HPV) is the most common sexually transmitted infection and the most common cause of cervical cancer and cervical cancer-related deaths in the United States (US).^1^–^4^ HPV infection and HPV-associated cervical cancer are preventable due to the advent of highly effective vaccines available since 2006. The Centers for Disease Control and Prevention (CDC), as well as the Advisory Committee on Immunization Practices, recommend the HPV vaccination series for girls aged 11 or 12 years of age, as well as females aged 13 to 26 not adequately vaccinated previously.^1^,^5^ In response to these recommendations, HPV awareness and vaccination rates have increased, but overall vaccination uptake remains well below national goals.^6^

Most alarming, significant disparities in HPV vaccine knowledge and vaccine series completion exist among those with the highest rates of HPV infection, including racial minorities, the under- and uninsured, and the economically disadvantaged.^7^–^11^ Further, cervical cancer incidence and mortality are higher among Black and Hispanic women as compared to White women, and cervical cancer incidence has been shown to be nearly twice as high among those living in poverty.^12^ For these reasons, it is a national priority to raise HPV awareness and vaccination among these disproportionately affected populations.

Due to challenges accessing primary care, disparate populations may benefit from engagement in HPV awareness and screening in alternative venues.^13^ Emergency departments (ED) have previously been demonstrated to be high-yield and effective venues for raising awareness of similar silent, stigmatizing, and deadly conditions–specifically, human immunodeficiency virus (HIV) and hepatitis C virus (HCV).^14^,^15^ Thus, it has been suggested that the ED be considered as an alternative setting for HPV education, screening, and vaccine administration in an effort to overcome barriers to HPV provision in all populations.^16^

We sought to measure and characterize HPV and cervical cancer awareness, risk factors, and HPV vaccination history among adult females presenting to an urban, academic ED. We hypothesized that female patients in the ED would demonstrate lower rates of HPV awareness and vaccination uptake than the general population.

## METHODS

We performed a cross-sectional, survey-based study at the University of Alabama at Birmingham (UAB), an urban, academic ED in the Southeast US with an affiliated emergency medicine residency program and an annual volume of approximately 65,000, from June 1–August 31, 2016. The UAB Institutional Review Board approved the protocol. Adult female patients, aged 18–32, were recruited to participate in the survey during their ED visit. We selected the age of 32 as the upper age limit for survey engagement to align with the availability of the HPV vaccine and vaccine administration guidelines beginning in 2006.^17^ Trained female research assistants (RA) recruited applicable patients and administered the survey Monday through Friday, 9 am to 4 pm (a convenience sample).

We collected 81 completed surveys in this pilot study to demonstrate feasibility. Exclusion criteria included the following: individuals who did not speak English or for whom communication barriers were present (eg, altered mental status, dementia); were medically or psychiatrically unstable; or who were undergoing active emergent evaluation or treatment; and individuals presenting for sexual assault. Subjects were recruited and administered the surveys in private rooms, and written informed consent was provided. RAs verbally delivered the survey questions in a casual interview style.

The 21-item survey instrument was developed by the study authors with the intent to be expansive, capturing general HPV awareness as well as HPV vaccine uptake information, in addition to demographic factors to include pre-existing cervical-cancer risk factors. While created de novo, the survey was influenced by previously published HPV awareness and attitude survey studies.^12^,^16^,^18^,^19^ The final survey (Appendix A) contained the following: demographic information; HPV infection and vaccine awareness; HPV vaccination history; HPV infection and cervical cancer risk factors. Survey questions met a SMOG (Simple Measure of Gobbledygook) reading index of 7th grade and a Gunning Fog score of 8.6, designating it “fairly easy to read.”^20^,^21^ The RA was allowed to clarify for the respondent any real-time questions regarding definitions and/or survey question verbiage as needed.

**Population Health Research Capsule**

What do we already know about this issue?

Human papillomavirus (HPV) is the most common sexually transmitted infection in the US, responsible for over 90% of cervical cancer cases. HPV vaccine uptake is low.

What was the research question?

Evaluate and characterize HPV awareness and vaccine rates in female patients presenting to the emergency department (ED).

What was the major finding of the study?

The female ED population represents a unique, at-risk cohort in regard to HPV and cervical-cancer risk factors.

How does this improve population health?

This study challenges emergency physicians to consider an expanded role as HPV educator and vaccine advocate, particularly for “at-risk” patients.

We selected a $20,000 annual household income threshold to reflect the 2016 Federal Poverty Line (FPL), the minimum amount of gross income that a family needs for food, clothing, transportation, shelter, and other necessities, as determined by the Department of Health and Human Services.^23^ In 2016, the FPL for a two-person household was $16,020 and for a three-person household was $20,160. To effectively capture the majority of respondents and understand the effect of socioeconomic status, we selected a delineation of $20,000 for the purpose of this study.

We collected the project data in a secure REDCap database.^22^ We determined the proportion of participants acknowledging HPV awareness and prior vaccination stratified by age, gender, race, access to primary care, income, and medical insurance status, assessing non-random differences of association using Fisher’s exact test (STATA/IC, 15.1, College Station, Texas).

## RESULTS

A total of 81 surveys were completed. Participant demographics are displayed in Table 1. Insurance status information was unavailable for one respondent (0.05%); likewise, income information was unavailable for five respondents (6.2%), and primary care provider status was unavailable for one respondent (1.2%). Three respondents (3.7%) identified more than one locale where they routinely sought primary care services.

Selected survey question responses with demographic delineation are displayed in Table 2. Survey response data regarding HPV vaccination rates was available for 80 survey respondents. One respondent who had previously received the HPV vaccine and four who had not received the vaccine did not disclose or were unaware of their annual household income.

HPV awareness was significantly higher among White respondents (96.4%, *p* = .016) and those with higher annual income (96.9%, *p* = .020). Among respondents with awareness of HPV, however, additional understanding of its designation as a common sexually transmitted infection and its association with cervical cancer was generally mixed. White patients were more likely to be aware of the HPV-cervical cancer association (). HPV vaccine awareness was higher among patients with an established primary care provider (84.6%, p = .003) and lower among those publicly insured or uninsured (42.2%, *p* = .034) and those with lower annual household income (40.9%, *p* = .048).

Only 38.8% respondents reported ever being offered the HPV vaccine and 24.7% previously completed the vaccination series. Of those vaccinated for HPV, 78% reported receiving the vaccine at their primary care physician’s office. Notably, 12.5% of respondents reported declining a prior HPV vaccination.

Of those age-eligible (n = 43) for HPV vaccination, 76% reported they would be comfortable initiating and receiving HPV vaccination in the ED.

A number of known cervical-cancer risk factors were also identified among respondents: inconsistent use of barrier contraceptive during sexual activity (59.5%); tobacco use (27.2%); pregnancy prior to age 17 (12.3%); and a family history of cervical cancer (7.6%) (Appendix C).

## DISCUSSION

In general, our survey respondents demonstrated a population with low HPV awareness and low HPV vaccination rates along with significant risk factors for HPV exposure and cervical cancer. The HPV vaccine series completion rate reported in this unique ED population was far below national vaccination rates. In 2017, 49% of US teens had completed the HPV vaccine series as compared to less than a quarter of our survey respondents.^24^ Racial and social inequities that have previously been associated with decreased vaccination rates in the primary care setting were echoed and, in some cases, amplified in this ED venue.^7^–^11^

In addition, it is alarming to note the number of vaccine-eligible women who reported they had never been counselled about nor offered the HPV vaccine, potentially because they did not have a primary care physician or the resources to see one regularly. Equally concerning was the nearly 13% refusal rate by those who had been offered the vaccine. Reasons given for declination were variable but predominantly included “I feel the illness is not severe or ‘bad enough’ to warrant vaccination,” and “The preservatives in the vaccines are dangerous.” This may represent lack of an established or primary care relationship to offer sufficient education regarding HPV, HPV’s direct association with cervical cancer, and vaccines in general.^25^

The study population also demonstrated a low awareness of HPV’s direct correlation with cervical cancer along with a significant risk profile for cervical cancer. Thirty-five percent were unaware of the HPV-cervical cancer connection, with even higher percentages noted among Black patients (44.9%) and the uninsured (57.1%). This has been demonstrated previously and may represent the influence of socioeconomic determinants of health, which can affect primary care access and utilization of preventive health services.^11^,^12^,^19^ Likewise, prevalence of several cervical-cancer risk factors among respondents was noted to be high as compared to the general population including smoking tobacco (27.2% among respondents compared to 15.5% nationwide) and full-term pregnancy before age 17 (12.3% of respondents compared to 19 births per 1000 in the US in 2017, or nearly seven times the national rate).^26^,^27^ The over-representation of cervical-cancer risk factors in this population underscores a vulnerable, at-risk subset.

The survey results highlight several potential engagement opportunites for emergency physicians (EP) to consider, including HPV awareness campaigns and ED-initiated HPV vaccine administration. Physician recommendation has been shown to be the most effective indicator of HPV uptake among vaccine-eligible women and may be equally effective in an ED setting.^28^–^31^ However, as Hill and Okugo demonstrated when assessing EP attitudes toward proposed HPV vaccine initiation in the ED, perceived barriers to HPV education and vaccine initiation in the ED setting do exist, including concerns regarding time requirements and reimbursement.^32^ Likewise, it is anticipated that some EPs may not embrace a preventative medicine role, a wheelhouse that traditionally has belonged to primary care physicians.

EPs, however, as demonstrated by our survey responses, may represent the only healthcare encounters many women ever have. EDs, as part of the national public health safety net, frequently encounter subsets of the general population who often have minimal interaction with or limited access to the traditional primary care-based healthcare system.^13^,^33^ Considering an expanded role for the EP, incorporating social determinants of health and their influence on our patients, beyond immediate stabilization and acuity, will be paramount to our patients’ health as well as general public health.

Initiating a conversation with EPs and primary care partners and/or community resources may be an important consideration as the healthcare system attempts to address HPV from a public health perspective.^34^ Certainly it is interesting to note that the majority of survey respondents were amenable to HPV vaccine initiation in the ED.

The ED setting has more recently been used as a venue for universal screening of other sexually transmitted viral infections, including HIV and HCV. Well established, opt-out screening programs of this type have resulted in the successful linkage of care of previously undiagnosed and untreated HIV- and/or HCV-positive patients, many of whom may have had no other access to testing or definitive care.^14^,^15^ Initiating a similar ED-based HPV vaccine awareness and initiation with a linkage to care program may be a way to bridge the existing HPV gaps in the US, particularly among racial and socioeconomic groups suffering from healthcare disparities.

Future, more expansive studies, considering additional evaluation of the adolescent age range as well as male patients, would be useful to further characterize the at-risk population presenting routinely to the ED setting.

## LIMITATIONS

This is a single-center study, and as such, results may not be universally applicable to all ED settings. In addition, the results represent a convenience sample collected as a pilot study and may not reflect overall ED demographics. In certain instances (eg, Hispanic respondents), small response numbers may limit data interpretation. Likewise, demographic data on excluded patients, as well as those who refused participation, was not collected in this feasibility study, which may also limit its general applicability. Exclusion of population subsets, particularly non-English speaking individuals, may result in skewed data; however, the majority of non-English speaking patients presenting to our ED represent underserved minorities who face similar healthcare inequities to other minority groups, including decreased rates of HPV vaccination. Thus, we suspect that inclusion of their responses and data would likely contribute to disparities noted in the discussion above. Because survey answers were self-reported by respondents, there was a potential for recall bias and/or social desirability bias, which could have contributed to over- or under-inflation of personal medical history and/or risk factor reporting in particular. Finally, pediatric patients, an important patient subset, were excluded from our study. A nearby children’s hospital caters to the majority of local pediatric emergency patients, limiting pediatric patient exposure in the study ED.

## CONCLUSION

HPV awareness and vaccination rates in this female ED population were low while cervical-cancer risk factor rates were high, identifying a particularly vulnerable population. EDs may be a high-yield venue for HPV and cervical cancer prevention including education, screening, and vaccine initiation.

## Supplementary Information







## Figures and Tables

**Table 1:** Demographics of participants who completed a survey on human papillomavirus awareness.

Race	n = 81
Black or African American	50 (61.7%)
White or Caucasian	28 (34.6%)
Hispanic or Latina	3 (3.7%)
Insurance Status	n = 80
Private	36 (45.0%)
Publicly insured or uninsured	44 (55.0%)
Income	n = 76
<$20,000/year	44 (57.9%)
>$20,000/year	32 (42.1%)
Primary Care Provider Status	n = 80
Established primary care	42 (52.5%)
Federally funded or “free” clinic	20 (25.0%)
Emergency department or urgent care	17 (21.25%)
None/other	5 (6.25%)

**Table 2:** HPV* and HPV vaccine awareness and uptake.

	Yes n (%)	No n (%)	p-value
Have you heard of HPV?	69 (85.2)	12 (14.8)	

Race			
Black or African American	41 (82.0)	9 (18.0)	
White or Caucasian	27 (96.4)	1 (3.6)	
Hispanic or Latina	1 (33.3%)	2 (66.7%)	.016
Insurance Status			
Private insurance	34 (94.4)	2 (5.6)	
Publicly Insured or Uninsured	35 (77.8)	10 (22.2)	.057
Income			
<$20,000/year	34 (77.3)	10 (22.7)	
>$20,000/year	31 (96.9)	1 (3.1)	.020
Primary Care Provider Status			
Established PCP	37 (94.9)	2 (5.1)	
Federally funded or “free” clinic	14 (73.7)	5 (26.3)	
ED or Urgent Care	12 (80.0)	3 (20.0)	
None or “Other”	4 (80.0)	1 (20.0)	0.072

Respondents who have received partial or complete HPV vaccinations	23 (28.75)	57 (71.25)	

Race	23 (28.75)	57 (71.25)	
Black or African American	13 (26.0)	37 (74.0)	
White or Causcasian	10 (37.0)	17 (63.0)	
Hispanic or Latina	0 (0)	3 (100)	.437
Insurance Status			
Private insurance	13 (36.1)	23 (63.9)	
Publicly insured or uninsured	10 (22.7)	34 (77.3)	.221
Income			
<$20,000/year	9 (20.9)	34 (79.1)	
>$20,000/year	13 (40.6)	19 (59.3)	.077
Primary Care Provider Status			
Established PCP	15 (35.7)	27 (64.3)	
Federally funded or “free” clinic	2 (10.0)	18 (90.0)	
ED or Urgent care	5 (29.4)	12 (70.6)	
None or “Other”	1 (14.3)	6 (85.7)	.150

* *HPV*, human papillomavirus; *PCP*, primary care provider; *ED*, emergency department.
