# Supplementary material for: Human Papillomavirus Awareness, Vaccine Status, and Risk Factors in Female Emergency Patients
Source: West J Emerg Med. 2020 Feb 24;21(2):203–8. doi: 10.5811/westjem.2019.12.44422 (PMC7081844; doi:10.5811/westjem.2019.12.44422)
Supplement: Supplementary file 1 [file wjem-21-203-s001.docx]

**Appendix A:** HPV Questionnaire

1. Have you heard of Human Papillomavirus or HPV?
2. Are you aware that HPV is the most common sexually transmitted infection in the U.S?
3. Are you aware that cancer-causing types of HPV are the primary cause of cervical cancer in women?
4. Are you aware that there are vaccines available for females aged 11-26 for the prevention of HPV infection?
5. Are you aware that HPV vaccines can effectively eliminate one’s risk of HPV-associated cancers?
6. Are you aware that the Advisory Committee on Immunization Practices, a division of the Center for Disease Control (CDC), recommends routine HPV vaccination administration for females aged 11-26?
7. Have you ever been offered the vaccine for HPV?
   1. Did you receive any part of the HPV vaccine?
   2. Do you complete the vaccine series (3-part)?
   3. Where do you receive the vaccine? (PCP’s office, Health department, other)
8. Have you ever declined the HPV vaccine? (please provide a reason; may provide more than one answer)
   1. I feel the illness is too rare to consider vaccination.
   2. I feel the illness is not severe or ‘bad enough’ to warrant vaccination.
   3. Vaccines cause autism.
   4. Vaccines have side effects.
   5. The preservatives in the vaccines are dangerous.
   6. Vaccinations represent a conspiratorial agenda from pharmaceutical companies and/or the government.
   7. I do not trust my care provider’s recommendations.
   8. I could not afford the vaccination.
9. Have you ever been told that you have or are infected with HPV?
10. Have you ever been told that you have ‘precancerous’ lesions on your cervix or cervical dysplasia (NOT cervical cancer)?
11. Have you ever been told that you have cervical cancer?
12. If/when sexually active, do you consistently use barrier contraceptives such as condoms?
13. Do you use oral contraceptive pills (OCPs)?
    1. Have you been on OCPs for 5 years or more?
14. Do you have a family history (mother or sister) of cervical cancer?
15. Have you ever been told you have HIV or AIDS?
16. Have you ever been told that you are immunosuppressed, excluding from HIV/AIDS (e.g. hematologic malignancy, immunosuppressive medications)?
17. Have you had three or more full-term pregnancies?
18. Have you had a previous full-term pregnancy before the age of 17?
19. Do you currently smoke tobacco?
20. If you were offered HPV testing, done by a cervical swab during a pelvic exam which was already being performed as part of your Emergency Department evaluation, would you accept?
21. (If respondent is <26 years of age) If initiation/starting of the HPV vaccination series was offered to you during your ED visit, would you accept?
