# Supplementary material for: Human Papillomavirus Awareness, Vaccine Status, and Risk Factors in Female Emergency Patients
Source: West J Emerg Med. 2020 Feb 24;21(2):203–8. doi: 10.5811/westjem.2019.12.44422 (PMC7081844; doi:10.5811/westjem.2019.12.44422)
Supplement: Supplementary file 2 [file wjem-21-203-s002.docx]

**Appendix B**: Additional Survey Response Data – HPV and HPV Vaccine Awareness

| **If heard of HPV, then aware that HPV is most common STI in US?** | **Yes**  **38 (55.1)** | **No**  **31 (44.9)** | ***p*-value** |
| --- | --- | --- | --- |
| **Race:** |  |  |  |
| Black or African American | **21 (51.2)** | **20 (48.8)** |  |
| White or Caucasian | **16 (59.3)** | **11 (40.7)** |  |
| Hispanic or Latina | **1 (100)** | **0 (0)** | **.703** |
| **Insurance Status:** |  |  |  |
| Private insurance | **21 (61.8)** | **13 (38.2)** |  |
| Publicly insured or uninsured | **17 (48.6)** | **18 (51.4)** | **.336** |
| **Income:** |  |  |  |
| <$20,000/year | **19 (55.9)** | **15 (44.1)** |  |
| >$20,000/year | **17 (54.8)** | **14 (45.2)** | **1.000** |
| **Primary Care Provider Status:** |  |  |  |
| Established PCP | **20 (54.1)** | **17 (45.9)** |  |
| Federally funded or ‘Free’ clinic | **8 (47.1)** | **9 (52.9)** |  |
| ED or Urgent Care | **7 (58.3)** | **5 (41.7)** |  |
| None or ‘Other’ | **3 (50.0)** | **3 (50.0)** | **.950** |
| **If heard of HPV, then aware that HPV is the primary cause of cervical cancer?** | **Yes**  **44 (64.7)** | **No**  **24 (35.3)** |  |
| **Race:** |  |  |  |
| Black or African American | **43 (55.1)** | **35 (44.9)** |  |
| White or Caucasian | **51 (71.8)** | **20 (28.2)** | **.042** |
| **Insurance Status:** |  |  |  |
| Private insurance | **24 (72.7)** | **9 (27.3)** |  |
| Publicly insured or uninsured | **20 (57.1)** | **15 (42.9)** | **.211** |
| **Income:** |  |  |  |
| <$20,000/year | **21 (61.8)** | **13 (38.2)** |  |
| >$20,000/year | **20 (66.7)** | **10 (33.3)** | **.796** |
| **Primary Care Provider Status:** |  |  |  |
| Established PCP | **26 (70.3)** | **11 (29.7)** |  |
| Federally funded or ‘Free’ clinic | **6 (46.2)** | **7 (53.8)** |  |
| ED or Urgent Care | **8 (66.7)** | **4 (33.3)** |  |
| None or ‘Other’ | **4 (66.7)** | **2 (33.3)** | **.488** |
| **HPV vaccine awareness (Is the patient aware a vaccine exists for HPV?)** | **Yes**  **55 (67.9)** | **No**  **26 (32.1)** |  |
| **Race:** |  |  |  |
| Black or African American | **31 (62.0)** | **19 (38.0)** |  |
| White or Caucasian | **23 (82.1)** | **5 (17.9)** |  |
| Hispanic or Latina | **1 (33.3)** | **2 (66.7)** | **.070** |
| **Insurance Status:** |  |  |  |
| Private insurance | **29 (80.6)** | **7 (19.4)** |  |
| Publicly insured or uninsured | **26 (57.8)** | **19 (42.2)** | **.034** |
| **Income:** |  |  |  |
| <$20,000/year | **26 (59.1)** | **18 (40.9)** |  |
| >$20,000/year | **26 (81.25)** | **6 (18.75)** | **.048** |
| **Primary Care Provider Status:** |  |  |  |
| Established PCP | **33 (84.6)** | **6 (15.4)** |  |
| Federally funded or ‘Free’ clinic | **7 (36.8)** | **12 (63.2)** |  |
| ED or Urgent Care | **10 (66.7)** | **5 (33.3)** |  |
| None or ‘Other’ | **5 (71.4)** | **2 (28.6)** | **0.003** |

*HPV,* Human papillomavirus; *STI*, sexually transmitted infection; *PCP*, primary care provider; *ED,* emergency department.
