# Supplementary material for: Human Papillomavirus Awareness, Vaccine Status, and Risk Factors in Female Emergency Patients
Source: West J Emerg Med. 2020 Feb 24;21(2):203–8. doi: 10.5811/westjem.2019.12.44422 (PMC7081844; doi:10.5811/westjem.2019.12.44422)
Supplement: Supplementary file 3 [file wjem-21-203-s003.docx]

**Appendix C**: Survey Data, Cervical Cancer Risk Factors

| **If/when sexually active, do you consistently use barrier contraceptives such as condoms?** | n = 79 |
| --- | --- |
| Yes | 28 (35.4%) |
| No | 47 (59.5%) |
| Never sexually active | 4 (5.1%) |
| **If you have ever used oral contraceptives (OCPs), have you been on OCPs for 5 years or more?** | n = 13 |
| Yes | 6 (46.2%) |
| No | 7 (53.8%) |
| **Do you have a family history (mother or sister) of cervical cancer?** | n = 79 |
| Yes | 6 (7.6%) |
| No | 73 (92.4%) |
| **Have you ever been told you have HIV or AIDS?** | n = 81 |
| Yes | 0 (0%) |
| No | 81 (100%) |
| **Have you ever been told that you are immunosuppressed, excluding HIV/AIDS (e.g. hematologic malignancy, immunosuppressive medications)?** | n = 81 |
| Yes | 7 (8.6%) |
| No | 74 (91.4%) |
| **Have you had three or more full-term pregnancies?** | n = 81 |
| Yes | 9 (11.1%) |
| No | 72 (88.9%) |
| **Have you had a previous full-term pregnancy before the age of 17?** | n = 81 |
| Yes | 10 (12.3%) |
| No | 71 (87.7%) |
| **Do you currently smoke tobacco?** | n = 81 |
| Yes | 22 (27.2%) |
| No | 59 (72.8%) |

*OCP*, oral contraceptive; *HIV*, human immunodeficiency virus; *AIDS*, Acquired Immune Deficiency Syndrome.

*N values less than 81 represent missing data due to respondent question deferment. In the specific case of OCPs, only 13 respondents total reported prior use.
